# Infection induced SARS-CoV-2 seroprevalence and heterogeneity of antibody responses in a general population cohort study in Catalonia Spain

**DOI:** 10.1038/s41598-021-00807-4

**Published:** 2021-11-03

**Authors:** Marianna Karachaliou, Gemma Moncunill, Ana Espinosa, Gemma Castaño-Vinyals, Alfons Jiménez, Marta Vidal, Rebeca Santano, Diana Barrios, Laura Puyol, Anna Carreras, Leonie Mayer, Rocío Rubio, Beatriz Cortés, Vanessa Pleguezuelos, Cristina O’Callaghan-Gordo, Serena Fossati, Ioar Rivas, Delphine Casabonne, Martine Vrijheid, Luis Izquierdo, Ruth Aguilar, Xavier Basagaña, Judith Garcia-Aymerich, Rafael de Cid, Carlota Dobaño, Manolis Kogevinas

**Affiliations:** 1 Barcelona Institute for Global Health (ISGlobal), 08036 Barcelona, Spain; 2 Centro de Investigação em Saúde de Manhiça (CISM), Maputo, Mozambique; 3 Centro de Investigación Biomédica en Red de Epidemiología y Salud Pública (CIBERESP), 08036 Madrid, Spain; 4 Genomes for Life-GCAT Lab, Institute for Health Science Research Germans Trias i Pujol (IGTP), Badalona, Spain; 5 Banc de Sang i Teixits (BST), Barcelona, Spain; 6 Universitat Pompeu Fabra (UPF), Barcelona, Spain; 7 Faculty of Health Sciences, Universitat Oberta de Catalunya, Barcelona, Spain; 8 Unit of Molecular and Genetic Epidemiology in Infections and Cancer (UNIC-Molecular), Cancer Epidemiology Research Programme, IDIBELL, Catalan Institute of Oncology, Hospitalet De Llobregat, Spain; 9 Hospital del Mar Medical Research Institute (IMIM), 08003 Barcelona, Spain

**Keywords:** Viral infection, Epidemiology, Risk factors

## Abstract

Sparse data exist on the complex natural immunity to SARS-CoV-2 at the population level. We applied a well-validated multiplex serology test in 5000 participants of a general population study in Catalonia in blood samples collected from end June to mid November 2020. Based on responses to fifteen isotype-antigen combinations, we detected a seroprevalence of 18.1% in adults (n = 4740), and modeled extrapolation to the general population of Catalonia indicated a 15.3% seroprevalence. Antibodies persisted up to 9 months after infection. Immune profiling of infected individuals revealed that with increasing severity of infection (asymptomatic, 1–3 symptoms, ≥ 4 symptoms, admitted to hospital/ICU), seroresponses were more robust and rich with a shift towards IgG over IgA and anti-spike over anti-nucleocapsid responses. Among seropositive participants, lower antibody levels were observed for those ≥ 60 years vs < 60 years old and smokers vs non-smokers. Overweight/obese participants vs normal weight had higher antibody levels. Adolescents (13–15 years old) (n = 260) showed a seroprevalence of 11.5%, were less likely to be tested seropositive compared to their parents and had dominant anti-spike rather than anti-nucleocapsid IgG responses. Our study provides an unbiased estimate of SARS-CoV-2 seroprevalence in Catalonia and new evidence on the durability and heterogeneity of post-infection immunity.

## Introduction

Severe acute respiratory syndrome coronavirus 2 (SARS-CoV-2) surveillance based on diagnostic testing, incomplete screening of all possible infections and imperfect test sensitivity may lead to a domino-like effect resulting in significant underestimation of the number of coronavirus disease 2019 (COVID-19) cases^1^. The high proportion of asymptomatic cases distorts even more the picture of the pandemic^2^.

Seroepidemiological studies have emerged across the world in order to provide us with a better estimate of the proportion of the population previously infected (vaccine-induced immunity is distinguishable)^3^. Nonetheless, many of these studies target specific populations (e.g. health care workers, previous hospitalized COVID-19 patients), use not well validated laboratory methods^4^ and have been mainly geared toward studying IgG responses to only one antigen^5^ Multiplex serology may improve the diagnostic power of such studies given the considerable heterogeneity in antibody responses between individuals. In particular, the virus has several antigenic epitopes that are the target of antibodies but not everyone responds to the same antigens^6^. Additionally, detection of certain isotype responses depends on the time since infection^7–9^. Within days of symptom onset, specific immunoglobulins M (IgM) are detected and after a lag period strong immunoglobulins G (IgG) responses typically occur. Immunoglobulin A (IgA) reponses are detected almost concurrently to IgM or earlier. With time, attenuation of antibody levels is expected due to decay of immune responses and transition of immunoglobulin production from short to long-lived plasma cell; thus cut-offs for seropositivity should take into account levels of waning immunity^10,11^. Moreover, the magnitude and type of antibody response correlates with disease severity. For example, most studies show that seroresponses are higher in more severe cases^12,13^. Recent data also show that multiplex serology is better correlated with levels of protective immunity^14^.

Limited data exist on the trajectories of antibody responses to SARS-CoV-2 over time and the factors that determine their heterogeneity. Notably, most studies consider individuals hospitalized or at least requiring some outpatient treatment^9,15–20^. Describing the characteristics of an effective immune response, as such encountered by asymptomatics or those with mild infections, is valuable. Early data show that some antigen and/or isotype responses dominate among milder infections^8,19,20^. Children are also facing effectively the infection, and studies comparing immune responses between SARS-CoV-2 infected children and adults have already provided some insights^21,22^. Considering members of the same family may resolve further questions related to time of infection, genetics, and other shared environmental exposures.

Taking advantage of multiplex serology to SARS-CoV-2, we describe the presence and heterogeneity of antibody responses in a population of 13–93 years old participants of existing cohort studies in Catalonia up to mid-November 2020. Catalonia in northeast Spain, has been among the hardest-hit populations in Europe from COVID-19.

## Results

### SARS-CoV-2 seroprevalence

Among the 10,837 adult participants of the COVID-19 Cohorts in CATalonia (COVICAT) study, the 4740 (44%) who donated a blood sample for serological testing were more likely to have reported symptoms, not having been tested before, be of higher education and less likely to work in their usual workplace during confinement and be smokers before confinement compared to those who participated only with questionnaire data (Supplementary Resource 1). A blood sample was available for all adolescents.

Table 1 presents the seroprevalence of SARS-CoV-2 based on the serostatus of fifteen isotype-antigen combinations [three isotypes: IgM, IgA and IgG; five viral target antigens: spike full protein (S), S2 fragmnet (S2), receptor binding domain (RBD), nucleocapsid full protein (NFL) and nucleocapsid C-terminal region (NCt)]. Details on the contribution of each isotype-antigen combination in the overall serostatus are available in Supplementary Resource 2. The overall SARS-CoV-2 seroprevalence among adults was 18.1% (IgM 3.7%, IgA 14.6%, IgG 9.0%) while 11.6% had an undetermined status. The highest prevalences were observed for RBD IgG (8.0%), S IgG (7.4%), RBD IgA (7.1%) and S IgA (6.9%). Adult participants of the second sampling period (n = 1089, 23%) (median date: 30 October 2020, range 8 September-17 November 2020) were more likely to be seropositive compared to those of the first sampling period (median date: 19 July 2020, range 23 June–31 July 2020) (23.8% versus 16.4%) (Supplementary Resource 3). To extrapolate the study results to Catalonia’s adult population, we used raked weights to balance the study sample characteristics (age, sex, educational level, health region, smoking) to those of the total population (more details in methods). We estimated a seroprevalence of 15.3% in adults in Catalonia. Among the 260 adolescents (13–15 years old), 11.5% were seropositive and 7.3% had an undetermined status (Table 1).

**Table 1:** SARS-CoV-2 seroprevalence (overall, by isotype, by isotype-antigen combination) in adult and adolescent participants of the COVICAT study in Catalonia (sampling period: end June to mid November 2020).

	Adults (n = 4740)						Adolescents (n = 260)					
	Positive		Negative		Undetermined		Positive		Negative		Undetermined	
	n	%	n	%	n	%	n	%	n	%	n	%
Overall	858	18.1	3331	70.3	551	11.6	30	11.5	211	81.2	19	7.3
**By isotype**												
IgM	175	3.7	4392	92.7	173	3.6	3	1.2	250	96.2	7	2.7
IgA	694	14.6	3616	76.3	430	9.1	19	7.3	233	89.6	8	3.1
IgG	426	9.0	4134	87.2	180	3.8	24	9.2	225	86.5	11	4.2
**By isotype-antigen combination**												
IgM NFL	6	0.1	4694	99.0	40	0.8	0	0	260	100	0	0.0
IgM NCt	18	0.4	4674	98.6	48	1.0	0	0	258	99.2	2	0.8
IgM RBD	88	1.9	4552	96.0	100	2.1	1	0.4	253	97.3	6	2.3
IgM S	49	1.0	4601	97.1	90	1.9	0	0	259	99.6	1	0.4
IgM S2	20	0.4	4637	97.8	83	1.8	0	0	258	99.2	2	0.8
IgA NFL	140	3.0	4310	90.9	290	6.1	1	0.4	256	98.5	3	1.2
IgA NCt	129	2.7	4479	94.5	132	2.8	0	0	257	98.8	3	1.2
IgA RBD	338	7.1	4210	88.8	192	4.1	17	6.5	240	92.3	3	1.2
IgA S	325	6.9	4259	89.9	156	3.3	13	5.0	245	94.2	2	0.8
IgA S2	209	4.4	4201	88.6	330	7.0	4	1.5	255	98.1	1	0.4
IgG NFL	59	1.2	4473	94.4	208	4.4	2	0.8	246	94.6	12	4.6
IgG NCt	98	2.1	4481	94.5	161	3.4	5	1.9	245	94.2	10	3.8
IgG RBD	380	8.0	4308	90.9	52	1.1	21	8.1	237	91.2	2	0.8
IgG S	351	7.4	4307	90.9	82	1.7	21	8.1	233	89.6	6	2.3
IgG S2	104	2.2	4353	91.8	283	6.0	6	2.3	233	89.6	21	8.1

### Antibody levels in time since infection

**Figure 1:**
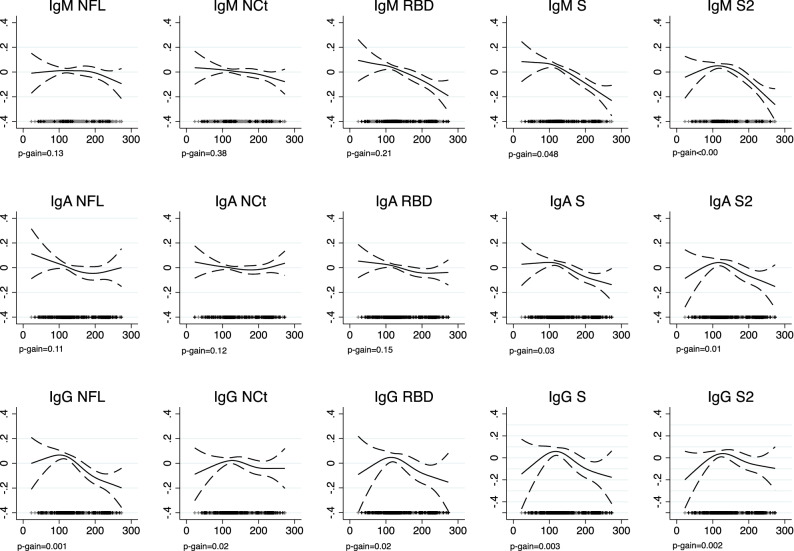
Generalized additive models for associations (95% CIs) of days since infection with antibody responses to the fifteen isotype-antigen combinations in seropositive participants of the COVICAT study. Plus symbols (+) represent overall seropositive participants and with grey color are participants seronegative to the specific isotype-antigen combination. For each isotype-antigen combination, levels are plotted irrespective of the serostatus to the specific combination.

We examined the association of time since infection with antibody levels using cross-sectional data from all seropositive adults with an estimated time since infection ranging from 23 to 273 days (Fig. 1). For each isotype-antigen combination, levels are plotted irrespective of the serostatus to the specific combination to not affect the heterogeneity of responses expected with time. We observed that RBD, S, and S2 IgM levels decreased significantly over time, with NFL and NCt IgMs being less affected; very few participants were actually seropositive to the specific isotype-antigen combinations. IgA responses to NFL, NCt and RBD sustained in time but those of IgA to S and S2 declined after around 120 days to lower levels. IgG responses seemed to be stable or peaking at around 100 days after infection and then started to decline rapidly for NFL IgG, modestly for RBD and S IgG, while NCt and S2 IgG levels were minimally affected. In a subgroup of 99 participants who were previously tested positive (self-reported result), 92% had a positive multiplex serology at a median of 102 days after first diagnosis (range 13–233 days) (Supplementary Resource 4).

### SARS-CoV-2 serology by COVID-19 symptoms

We present the distribution of symptoms among adults and adolescents by SARS-CoV-2 serostatus in Table 2. Among adults, all symptoms were more prevalent (p-value < 0.05) in SARS-CoV-2 seropositive versus seronegative participants with most remarkable differences seen for loss of odor/taste and fever. Among seropositives, 38.4% were asymptomatic and seropositives versus seronegatives were more likely to report ≥ 4 symptoms (28.5% vs 9.6%). The distribution of symptoms among seropositive adolescents was slightly different compared to adults, with a statistically significant higher proportion reporting chest pain (25% vs. 8% in adolescents and adults respectively), and a lower proportion reporting fever (16.7% vs. 30.6%) and respiratory symptoms (cough and dyspnea 25% vs. 39.1%). Having four or more symptoms, fatigue, chest pain, runny nose, loss of odor/taste and fever were statistically significant more prevalent in seropositive versus seronegative adolescents.

**Table 2:**
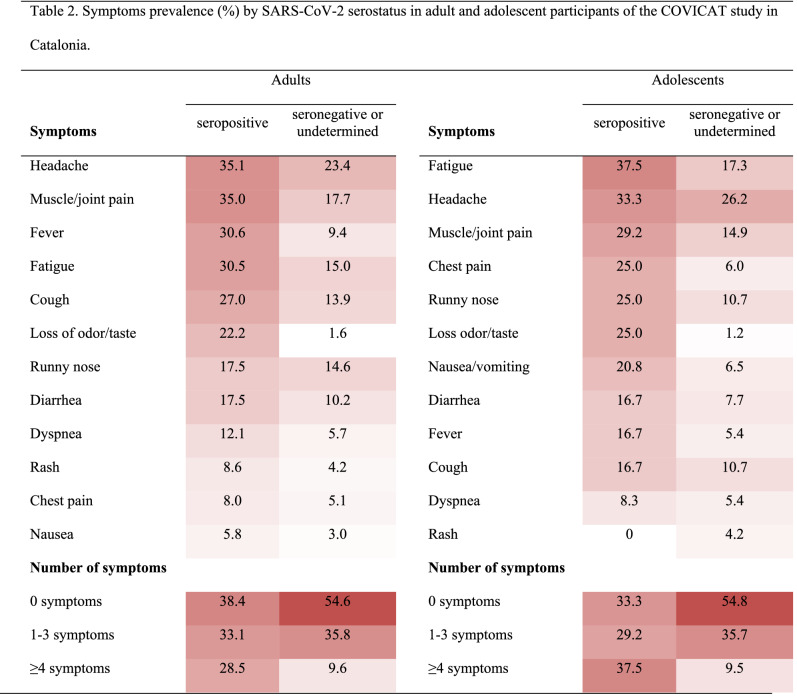
Symptoms prevalence (%) by SARS-CoV-2 serostatus in adult and adolescent participants of the COVICAT study in Catalonia.

Symptoms are sorted in adults and adolescents in decreasing frequency as observed among seropositives.

Darker red = higher prevalence.

Demographic and clinical characteristics of adult participants according to SARS-CoV-2 serostatus and severity of infection are presented in Supplementary Resource 5. Participants reporting contact with a COVID-19 case and non-smokers were more likely to be seropositive. The proportion of participants reporting contact with a COVID-19 case, being previously tested, reporting any chronic disease or being overweight/obese increased with the severity of infection.

### Antibody responses by the severity of infection

**Figure 2:**
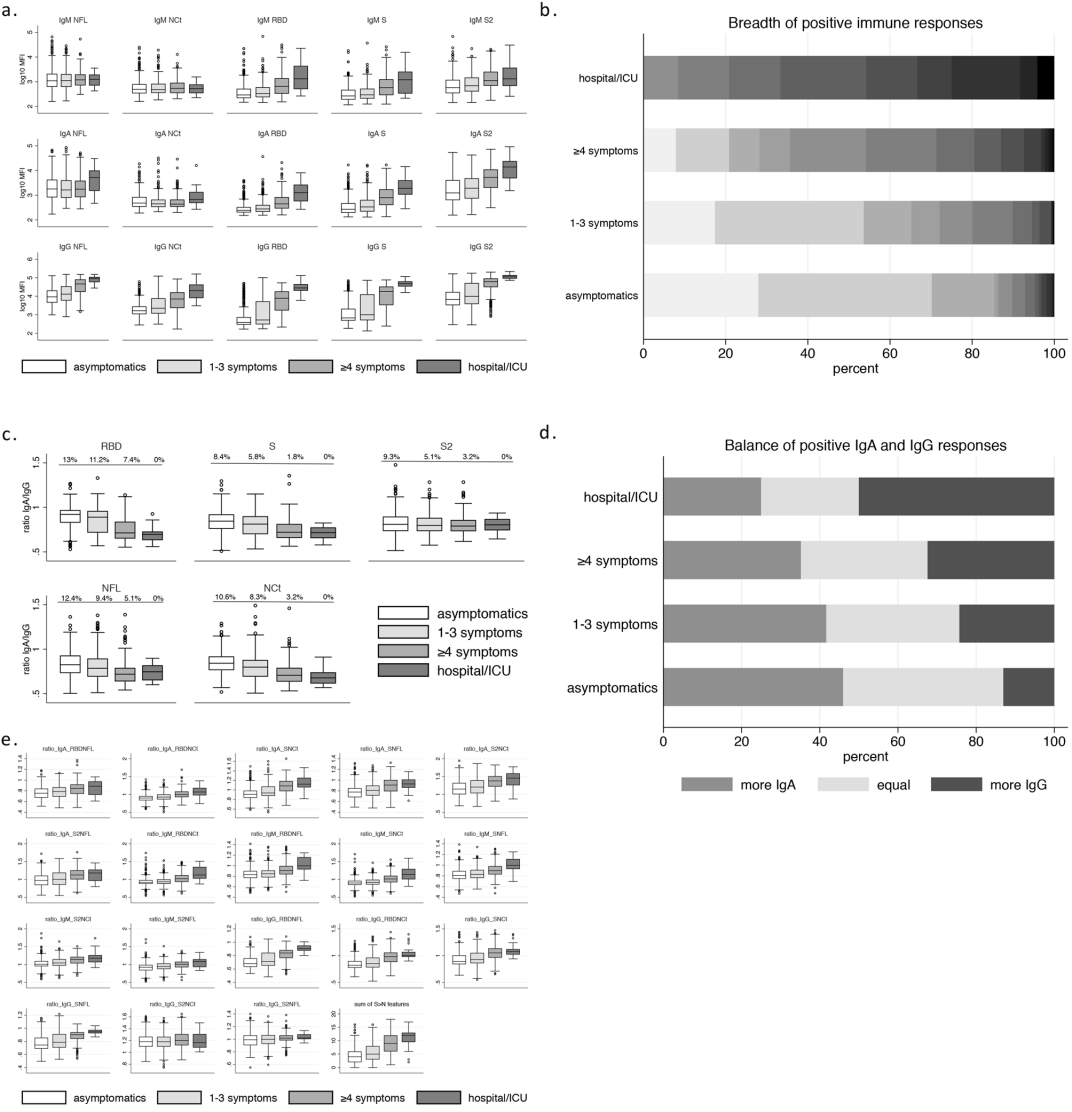
Differences in antibody responses according to the severity of infection among SARS-CoV-2 seropositive participants. Comparison of (**a**) antibody levels (log10-transformed values) of the fifteen isotype-antigen combinations, (**b**) breadth of positive immune responses ranging from 0 responses (light grey) to 13 responses (almost black), (**c**) ratio IgA/IgG antibody levels (log10-transformed values) for each of the antigens (the proportion of the participants showing ratio > 1 is listed in the top of each graph), (**d**) proportion of people showing more IgA, more IgG and equal number of positive IgA and IgG responses and (**e**) ratios of anti-spike (S, S2, RBD) over anti-nucleocapsid (NFL, NCt) responses for every combination and isotype. S for spike protein and N for nucleocapsid. Supplementary Resource 6 presents corresponding p-values.

To determine whether the severity of infection is associated with quantitatively and qualitatively different antibody responses we performed four analyses. Firstly, we compared antibody levels of the 15 isotype-antigen combinations between asymptomatic individuals (n = 322), those reporting 1–3 symptoms (n = 276), those reporting ≥ 4 symptoms (n = 216) and those admitted to hospital/ICU (n = 24) (Fig. 2a). We observed lower levels among asymptomatics and higher levels among those admitted to hospital/ICU (apart from NFL and NCt IgMs). Gradient differences were most evident among IgG to NFL and NCt and all RBD, S and S2 responses. Secondly, we found that the breadth of positive immune responses (aggregate number of seropositive isotype-antigen combinations), increased with severity of infection (Fig. 2b). Thirdly, we explored for differences in isotype responses. We observed a higher increase in levels of IgG than IgA responses with increasing severity (Fig. 2a). In Fig. 2c, using IgA/IgG ratios, we observed that IgA levels were closer to IgG levels among asymptomatics and more likely to exceed them compared to those with more severe infection. Also, we found that among asymptomatics, a higher proportion (46%) had more positive IgA than IgG responses compared to the 13% displaying more IgG than IgA responses (Fig. 2d). We observed reverse findings among those hospitalized. Finally, we compared responses related to spike protein versus nucleocapsid antigens. Experiencing a more severe infection was associated with a shift towards spike over nucleocapsid antibody responses (Fig. 2e). The overall trend is reflected in the last graph of Fig. 2e depicting differences in the number of features that had greater spike than nucleocapsid related responses (ratios over one). Because time since infection may impact the associations mentioned above, we repeated all analyses in two strata of seropositive individuals those sampled before and after 120 days since infection. Results were materially unchanged (data not shown).

### Antibody responses by age, sex, and lifestyle characteristics

We examined for differences in antibody levels and breadth of positive immune responses among seropositive adults with respect to age, sex, smoking, and body mass index (BMI) status before confinement (Table 3, Supplementary Resource 7). Participants 60 years of age or older had lower responses to almost all isotype-antigen combinations and a lower breadth of positive responses but had higher levels of NFL IgA. Females had statistically significant higher NFL, NCt and S2 IgM responses but lower NFL, NCt, and S2 IgA responses. Overweight or obese people had higher levels to almost all IgA and IgG responses and a higher breadth of positive responses. On the other hand, smokers displayed lower levels of almost all antibodies and a lower breadth of positive responses. We additionally adjusted for the severity of infection, considering it as a mediator of the associations. After adjustment, age ≥ 60 years old was associated with lower levels of IgM to S2 but higher levels of IgA to NFL. Associations with sex remained, with women showing an overall lower number of positive responses. Associations with overweight/obesity were largely diluted but remained positive. Smoking was consistently associated with lower levels and a lower breadth of positive responses. We repeated all the analyses excluding those seronegative for each isotype-antigen combination and results for smoking and BMI status were similar (Supplementary Resource 7).

**Table 3:**
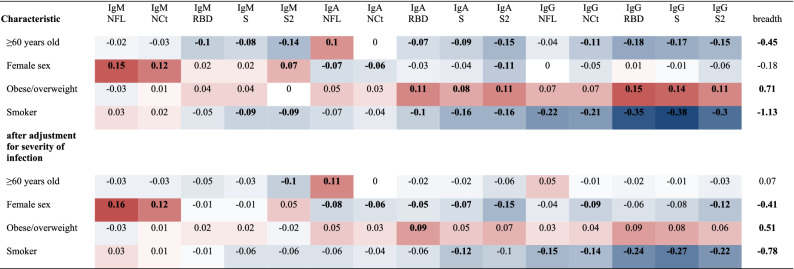
Adjusted associations (β) between each characteristic and antibody levels for each of the fifteen isotype-antigen combinations and the breadth of positive immune responses for SARS-CoV-2 seropositive participants, the COVICAT study.

Associations (β coefficients) of each listed characteristic with log_10_ transformed MFI values of each isotype-antigen combination adjusted for all the listed characteristics and days since infection. Bold indicates statistically significant associations (Supplementary Resource 7 presents corresponding p-values and 95% CI). The red color indicates a positive association while the blue color a negative association (associations with breadth are not comparable with those of antibody levels and thus not colored).

### Antibody responses among adolescents

**Figure 3:**
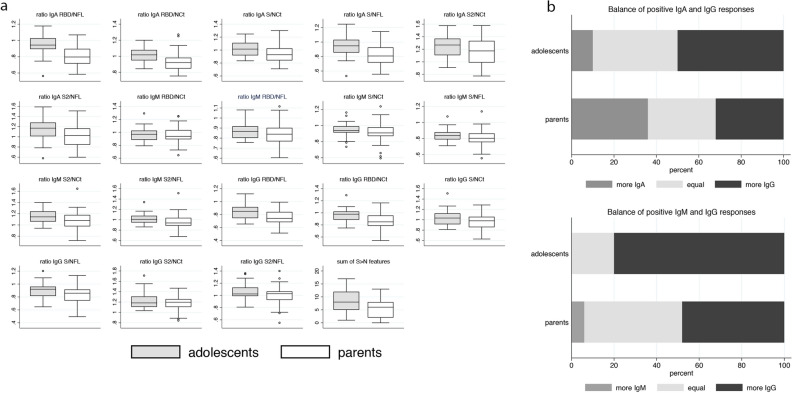
Differences in antibody responses between SARS-CoV-2 seropositive adolescents and parents. Comparison of antibody responses, (**a**) ratio of anti-spike (S, S2, RBD) over anti-nucleocapsid (NFL, NCt) responses for every combination and isotype and (**b**) proportion of people showing more IgA or IgM, more IgG and equal number of positive IgA or IgM and IgG responses.

Serological data among 260 parent–child pairs showed a much lower risk for seropositivity among adolescents (n = 30) than their parents (n = 50) [RR: 0.6, 95% CI 0.39–0.91)]. Sample collection took place at the same day for parents and their children. Among seropositive, adolescents had higher responses to S, S2 and RBD IgG, whereas parents had higher responses to NFL and NCt IgA (Supplementary Resource 8). The dominant IgG responses related to spike protein (S, S2, RBD) observed among adolescents were further confirmed when we compared the ratios of spike versus nucleocapsid responses (Fig. 3a) and the number of positive IgG compared to IgA or IgM responses (Fig. 3b). When we restricted our analysis to the 16 parent–child pairs that were both tested seropositive, we observed similar results although most were no longer statistically significant (data not shown).

## Discussion

The COVICAT study is one of the largest studies examining the complex natural immunity to SARS-CoV-2 at a population level. Based on multiplex serology testing of 5000 participants, we detected a SARS-CoV-2 seroprevalence of 18.1% in adults and much lower, of 11.5%, in adolescents. Additionally, 11.6% of adults and 7.3% of adolescents showed marginal seroresponses (undetermined). Severity of infection, determined the magnitude, breadth and specificity (towards certain antigens and/or isotypes) of immune responses long time after the acute phase of infection. We also identified diverse associations between individuals’ characteristics, including age, sex, smoking, and BMI status, with antibody responses.

Up to mid-November, there were 238,596 confirmed COVID-19 cases in Catalonia in people > 20 years old which corresponds to 3.9% of the population^23^. This proportion is much lower than our proportion of infected individuals based on serology (18.1%). We expected this difference, as surveillance systems are restricted by the emergency and load of testing clinically evident infections and high-risk individuals. Meanwhile, updated data from the fourth phase of a nationwide seroprevalence study in Spain (ENE-COVID) reported a seroprevalence of 9.9% in Spain and 11.6% in Catalonia until the end of November^24^. They used two tests, a point-of-care rapid test determining IgG against RBD and an immunoassay detecting IgG against nucleocapsid (not yet available for the fourth phase). Although the seroprevalence in ENE-COVID for Catalonia is lower than in our study it is not so different when compared with the seroprevalence for RBD and/or NCt IgG being 8.1% in our study. Thus, the difference in seroprevalence between the two studies could be partially attributable to the less extensive serological testing in ENE-COVID compared to our multiplex approach. Another argument for this scenario is that seroprevalence in adolescents, whose responses were primarily IgG anti-RBD, was not so different between the two studies (8.6% for 10–14 years of age in ENECOVID and 11.5% for 13–15 years old in our study). An increasing number of seroprevalence surveys now utilize multi-antigen and multi-isotype antibody responses because seroresponses might be skewed to different antigens and isotypes depending on clinical and individuals’ characteristics^16,25,26^. Also, the importance of IgA isotype in diagnostic accuracy of SARS-CoV-2 serological tests is emerging^7,17,27^.

We found no differences in seroprevalence between females and males or with age among adults. Interestingly, seropositive participants older than 60 years of age had higher NFL IgA levels and women had lower antibody levels and number of seropositive responses (apart from NFL and NCt IgM). Age and sex-specific antibody responses against SARS-CoV-2 have been documented but results are mixed^16,28,29^. Similar to other studies, seroprevalence among young adolescents was lower than among adults^30^. Within one family, adolescents were at lower risk for seropositivity compared to their parents. We cannot make direct conclusions about children’s role in transmitting SARS-CoV-2 within the household, but evidence argues for a reduced, marginal or conditional contribution^30,31^. It remains unclear why children are less susceptible to infection but mechanisms related to the number of ACE-2 receptors^32^, the naivety of innate immunity^33^ and preexisting human coronaviruses-elicited immunity^34^ are proposed.

A striking observation was that over 90% of previously tested positive participants had detectable antibodies up to 7 months after their first diagnosis, but most of them were either hospitalized or had experienced ≥ 4 symptoms. In the overall seropositive population of our study including infections of varying severity, we observed sustained levels for IgA and IgG responses at least 4 months after infection. More stable responses up to 9 months after infection were evident for NCt, RBD IgA and NCt, S2 IgG. We did not have repeated samples but a number of other studies did and showed limited loss of IgG antibodies and some loss of IgA antibodies over time^9,16,18,35–37^. More importantly, two recent studies showed that seropositive participants had a significantly decreased risk of re-infection up to 6 months after first infection^38,39^. It remains to be determined what levels of antibodies to what specific antigen epitopes protect people from recurrent infections.

Our findings are in agreement with previous reports showing that asymptomatics account for a significant proportion of the infected population (approximately 40%) and that a range of symptoms occurs with COVID-19 infection with the most specific being the loss of odor/taste and fever^40,41^. Importantly, the clinical spectrum of SARS-CoV-2 infection reflects a spectrum of antibody responses. With increasing severity of infection, we observed that hosts mounted more robust and rich responses. A recent study showed that the immune response of severely infected subjects was spread to subdominant viral antigens as well^42^. A novel finding in our study was that asymptomatics were more likely to have greater IgA than IgG responses compared to those experiencing more severe disease although the magnitude of IgA responses remained in lower levels in asymptomatics compared to those admitted to hospital/ICU. IgA could contribute to virus neutralization early in the infection to a greater extent compared with IgG^8^. Similar to us, most studies have described higher levels of antibodies among those with more severe disease, and some have suggested that a robust IgA response, in particular, may have a pathological role in SARS-CoV-2 infection^9,16,17,22^. Collectively with our data, it seems that IgA at low levels may be able to control the infection, but it could be associated with detrimental effects when boosted to higher levels along with other responses. In our study, we cannot disentangle to which extent the severity of infection drives these immune responses or whether these responses play a role in the pathogenesis of SARS-CoV-2.

Contradictory to two previous smaller studies, we found a shift towards spike over nucleocapsid responses with increasing severity of infection^19,20^. This discrepancy might be related to the fact that each study examined different immune features and we collected samples long after infection. Different rates of decay of anti-spike versus anti-nucleocapsid antibodies might have affected our results^43,44^. Although unclear, it is possible that the severity of infection determines the antibody production in the longterm (e.g. from long-lived plasma cells) in an antigen-specific manner^45^. For example, a recent study demonstrated that subjects with more severe disease mounted a larger memory B cell formation against the spike, but not the nucleocapsid^42^. Serological data from the group of adolescents point to very specific responses mainly of IgG against spike protein, consistent with previous studies^21,22^. We should note that children until late adolescence have a lower capacity of generating IgA^46^. These data suggest that adolescents use IgG alone to control the infection and that lack of anti-nucleocapsid responses might indicate a less widespread infection than adults. Such differences between adult and childhood immune responses should be delineated given the less harmful effects of SARS-CoV-2 infection in children.

Consistent with other studies, we detected higher levels of antibodies among overweight/obese participants. This association largely diluted when we adjusted for severity of infection, suggesting that higher levels were a consequence of the more severe disease experienced by obese people (adjustment by severity of infection was not considered in previous studies)^16,47^. In our population, the highest proportion of overweight/obese people was among those experiencing more severe infection. World Obesity Forum recently reported that COVID-19 mortality increased along with the countries’ prevalence of obesity, even after adjusting for age and wealth^48^. These data suggest that despite displaying robust serological responses, overweight/obese infected people are more likely to develop severe infection than non-overweight/obese people. Reduced levels of SARS-CoV-2 antibodies and breadth of immune responses were detected among seropositive smokers compared to non-smokers irrespective of the severity of infection. Two studies report similar results concerning levels of antibodies^16,49^. These data suggest that smokers present a weakened immune response to SARS-CoV-2. Thus, the higher COVID-19 morbidity among smokers might be due to impaired immunity as reflected in lower antibody levels^50^. Simultaneously, the paradox of low prevalence of smoking among SARS-CoV-2 infected people might be due to low/non-detectable levels of antibodies. Of course, this would not affect results from PCR tests but it has been suggested, although not investigated, that smoking might decrease nasopharyngeal viral load resulting more often in false-negative results^51^. We need more studies in this perspective.

We acknowledge that this is not a random population-based study as it recruited participants from pre-existing cohorts. On the other hand, existing cohorts allow us to quickly contact a population, achieve a high participation rate and access pre-pandemic information. The study primarily consisted of people 40–70 years old (≈ 90%), owing to the age distribution in original cohorts but we combined several cohorts in order to include younger and older people. Volunteer bias is always of concern, as we observed a lower participation rate for those who had received a test; this would have lead to underestimation of true seroprevalence. We assumed that all persons infected with SARS-CoV-2 have detectable antibodies at the time of sampling. The 8% of previously tested positive participants with a negative serology in our study might indicate the proportion of the population who lost immunity, were antibody non-responders or had false-positive results in the first test. Moreover, we could not verify whether the symptoms reported were attributable to a SARS-CoV-2 infection given the limited access of the population to diagnostic tests at the initial months of the pandemic.

## Conclusion

Collectively, the data presented here argue for a higher number of exposed individuals to SARS-CoV-2 in Catalonia, than what has been described, but still the majority of the population remains unexposed. Although further analysis with repeated samples will allow us to describe the progression of antibody levels in time, we observed that even 4–9 months after infection, responses against SARS-CoV-2 were evident. Individuals presented strikingly heterogeneous immune responses depending on the severity of infection. Factors such as obesity and smoking that are related to significant COVID-19 morbidity and mortality probably determine antibody responses.

## Methods

### Study design and setting

The COVICAT study includes participants from different pre-existing ongoing population-based cohorts in Catalonia and was developed following the COVID-19 pandemic. Eligible participants were from three adult population-based cohorts (GCAT, Genomes for life^52^; MCC-Spain, population controls from Catalunya^53^; and ECRHS, European Community Respiratory Health Survey^54^), two mother–child cohorts [INMA-Sabadell, (INfancia y Medio Ambiente)^55^ children born in 2005–2007 and their mothers; BiSC, Barcelona Life Study Cohort pregnant women recruited immediately before and during the pandemic] and two small general population cohorts in special populations (Urban Training, older persons; and LeRAgs, rural population). The eligible adult population consists of 19,424 people and we were able to contact 18,737 (96%) using email and telephone messages or calls. Of them, 10,837 (58%) participated in the study. Just after the strict first confinement period, we invited them to complete an online questionnaire about COVID-19 compatible symptoms, diagnostics as well as about occupational and financial aspects, sociodemographic and lifestyle characteristics, mental health and chronic diseases. We also did telephone interviews for those unfamiliar with the use of online approaches. Participants were then asked to donate a blood sample in different facilities. We offered the option for older people and those living in remote areas of collecting a blood fingerprick sample at their residence, as well as a second opportunity for donating a blood sample during September–November 2020 for all participants not able to provide a sample earlier. Participants of the second sampling period completed an additional questionnaire related to COVID-19 symptoms and testing in order to update the relative information from the main questionnaire. We also had available samples and questionnaire information on 260 adolescent participants of the INMA-Sabadell, cohort. Details on the sources and methods of assessment of variables are presented in the Supplementary Resource 9.

All participants gave written informed consent before participation in the study. For individuals younger than 18 years, parents or a legal representative provided consent. The study was approved by the Parc de Salut Mar Drug Research Ethical Committee (IBR number: 2020/9307/I). All methods were performed in accordance with the relevant guidelines and *regulations.*

### Serology

Blood samples were processed within 24 h of collection and were analyzed at the ISGlobal Immunology laboratory in Barcelona. The levels [median fluorescence intensity (MFI)] of IgG, IgM and IgA were assessed by high-throughput multiplex quantitative suspension array technology, including, as SARS-CoV-2 antigens, the S (aa 1–1213 expressed in Expi293 and His tag-purified) and the S2 fragment (purchased from SinoBiologicals), the RBD (donated by the Krammer lab, Mount Sinai, NY), the NFL and the specific NCt (expressed in *E. coli* and His tag-purified). Assay performance was previously established as 100% specificity and 95.78% sensitivity for seropositivity 14 days after symptoms onset^56^. Antigen-coupled microspheres were added to a 384-well μClear^®^ flat bottom plate (Greiner Bio-One, Frickenhausen, Germany) in multiplex (2000 microspheres per analyte per well) in a volume of 90 μL of Luminex Buffer (1% BSA, 0.05% Tween 20, 0.05% sodium azide in PBS) using 384 channels Integra Viaflo semi-automatic device (96/384, 384 channel pipette). Hyperimmune pools were used as positive controls prepared at twofold, 8 serial dilutions from 1:12.5. Pre-pandemic samples were used as negative controls to estimate the cut-off of seropositivity. Ten microliter of each dilution of the positive control, negative controls and test samples (prediluted 1:50 in 96 round-bottom well plates), were added to a 384-well plate using Assist Plus Integra device with 12 channels Voyager pipette (final test sample dilution of 1:500). To quantify IgM, test samples and controls were pre-treated with anti-Human IgG (Gullsorb) at 1:10 dilution, to avoid IgG interferences. Technical blanks consisting of Luminex Buffer and microspheres without samples were added in 4 wells to control for non-specific signals. Plates were incubated for 1 h at room temperature in agitation (Titramax 1000) at 900 rpm and protected from light. Then, the plates were washed three times with 200 μL/well of PBS-T (0.05% Tween 20 in PBS), using BioTek 405 TS (384-well format). Twenty five microliter of goat anti-human IgG-phycoerythrin (PE) (GTIG-001, Moss Bio) diluted 1:400, goat anti-human IgA-PE (GTIA-001, Moss Bio) 1:200, or goat anti-human IgM-PE (GTIM-001, Moss Bio) 1:200 in Luminex buffer were added to each well and incubated for 30 min. Plates were washed and microspheres resuspended with 80 μL of Luminex Buffer, covered with an adhesive film and sonicated 20 s on sonicator bath platform, before acquisition on the Flexmap 3D reader. At least 50 microspheres per analyte per well were acquired, and MFI was reported for each analyte. Assay positivity cut-offs specific for each isotype and analyte were calculated as 10 to the mean plus 3 standard deviations of log_10_-transformed MFI of 128 pre-pandemic controls. Results were defined as undetermined when the MFI levels for a given isotype-analyte were between the positivity threshold and an upper limit at 10 to the mean plus 4.5 standard deviations of the log_10_-transformed MFIs of pre-pandemic samples, and no other isotype-antigen combination was above the positivity cut-off.

### Statistics

Descriptive analyses of the study population characteristics were conducted. We used raked weights to extrapolate seroprevalence to the total population of Catalonia aged more than 20 years. Briefly, raking calculates weigths so that the weighted sample has the same marginal distribution than the reference population in terms of the variables used to calculate the weights^57^. In particular, we used the joint sex-age (in 10-year groups) distribution, educational level, health region and smoking. Population data were obtained from the National Statistics Institute and from the Catalan Health Survey^58^. The COVICAT study includes lower numbers of younger ages, which leads to overdispersed weights and gaps in the distribution of the weights. For this reason, we restricted the extremes weights by trimming the distribution at 99% of the weights. Generalized additive models were used to explore the shape of the relationship between days since infection and antibody levels to each of the fifteen isotype-antigen combination. Antibody levels were log_10_-transformed to normalise their distribution. Differences in antibody levels and ratios by severity of infection were examined using oneway Anova and pairwise comparisons were performed using Tukey post hoc-test. Differences in immune responses between adolescents and parents were examined with t-tests. Multivariable regression models were applied to examine among seropositive individuals the association between age, sex, smoking and BMI status before confinement (all in the same model adjusted also for days since infection) and log_10_-transformed antibody levels and the breadth of positive responses. All analyses were conducted using Stata version 16 (StataCorp LP, College Station, Texas).

## Supplementary Information

Supplementary Information.

## Abbreviations

SARS-CoV-2: Severe acute respiratory syndrome coronavirus 2
COVID-19: Coronavirus disease 2019
IgM: Immunoglobulin M
IgG: Immunoglobulin G
IgA: Immunoglobulin A
COVICAT: COVID-19 Cohorts in CATalonia
S: Spike full protein
S2: S2 fragmnet
RBD: Receptor binding domain
NFL: Nucleocapsid full protein
NCt: Nucleocapsid C-terminal region
ICU: Intensive Care Unit
BMI: Body mass index
INMA: INfancia y Medio Ambiente
MFI: Median fluorescence intensity
