# Supplementary material for: Infection induced SARS-CoV-2 seroprevalence and heterogeneity of antibody responses in a general population cohort study in Catalonia Spain
Source: Sci Rep. 2021 Nov 3;11:21571. doi: 10.1038/s41598-021-00807-4 (PMC8566562; doi:10.1038/s41598-021-00807-4)
Supplement: Supplementary file 1 — Supplementary Information. [file 41598_2021_807_MOESM1_ESM.docx]

| **Supplementary Resource 1.** Characteristics by participation or not with a blood sample for serological analysis in the COVICAT study (adults only). | | | | | |
| --- | --- | --- | --- | --- | --- |
|  | **Blood sample** | | | |  |
|  | no (n=6097) | | yes (4740) | |  |
| **Characteristic** | n | % | n | % | p-value |
| **Age** |  |  |  |  | 0.000 |
| 20-40 years old | 235 | 3.85 | 175 | 3.7 |  |
| 40-60 years old | 4309 | 70.7 | 3119 | 66.7 |  |
| 60-80 years old | 1514 | 24.8 | 1355 | 29.0 |  |
| 80-93 years old | 39 | 0.6 | 27 | 0.6 |  |
| Total | 6097 | 100 | 4676 | 100.0 |  |
| **Gender** |  |  |  |  | 0.825 |
| Male | 2346 | 38.5 | 1814 | 38.3 |  |
| Female | 3751 | 61.5 | 2926 | 61.7 |  |
| Total | 6097 | 100 | 4740 | 100 |  |
| **Ethninicy** |  |  |  |  | 0.000 |
| White / Caucasian | 5693 | 96.4 | 4306 | 97.7 |  |
| Other | 213 | 3.6 | 103 | 2.3 |  |
| Total | 5906 | 100.0 | 4409 | 100.0 |  |
| **Educational level** |  |  |  |  | 0.025 |
| primary or less | 764 | 12.5 | 511 | 10.9 |  |
| secondary | 2498 | 41.0 | 1884 | 40.3 |  |
| university | 2816 | 46.2 | 2265 | 48.5 |  |
| other | 12 | 0.2 | 12 | 0.3 |  |
| Total | 6090 | 100.0 | 4672 | 100.0 |  |
| **degree of urbanization** |  |  |  |  | 0.005 |
| city | 5078 | 89.1 | 3840 | 91.1 |  |
| town/suburb | 532 | 9.3 | 325 | 7.7 |  |
| rural area | 89 | 1.6 | 51 | 1.2 |  |
| Total | 5699 | 100.0 | 4216 | 100.0 |  |
| **Work during confinement** |  |  |  |  | 0.000 |
| No | 2298 | 38.1 | 1818 | 41.2 |  |
| Yes, teleworking | 1770 | 29.4 | 1385 | 31.4 |  |
| Yes, partially teleworking | 515 | 8.5 | 401 | 9.1 |  |
| Yes, in my usual place of work | 1447 | 24.0 | 811 | 18.4 |  |
| Total | 6030 | 100.0 | 4415 | 100.0 |  |
| **Contact with a COVID-19 case** |  |  |  |  | 0.035 |
| No | 3870 | 63.7 | 2999 | 65.6 |  |
| Yes | 1112 | 18.3 | 751 | 16.4 |  |
| Don't know | 1097 | 18.0 | 823 | 18.0 |  |
| Total | 6079 | 100.0 | 4573 | 100.0 |  |
| **Self-reported symptoms compatible with COVID-19** |  |  |  |  | 0.000 |
| 0 symptoms | 3643 | 59.8 | 2389 | 51.6 |  |
| 1-3 symptoms | 1808 | 29.7 | 1634 | 35.3 |  |
| 4 or more symptoms | 646 | 10.6 | 603 | 13.0 |  |
| Total | 6097 | 100.0 | 4626 | 100.0 |  |
| **SARS-COV-2 test before** |  |  |  |  | 0.001 |
| No | 4819 | 81.8 | 3791 | 84.3 |  |
| Yes | 1072 | 18.2 | 708 | 15.7 |  |
| Total | 5891 | 100.0 | 4499 | 100.0 |  |
| **SARS-CoV-2 test result** |  |  |  |  | 0.454 |
| Positive | 133 | 12.4 | 99 | 14.0 |  |
| Negative | 898 | 83.8 | 577 | 81.5 |  |
| Don't know | 41 | 3.8 | 32 | 4.5 |  |
| Total | 1072 | 100.0 | 708 | 100.0 |  |
| **Hospitalised due to COVID-19** |  |  |  |  | 0.588 |
| No | 5794 | 99.3 | 4452 | 99.4 |  |
| Yes | 43 | 0.7 | 29 | 0.6 |  |
| Total | 5837 | 100.0 | 4481 | 100 |  |
| **General health status** |  |  |  |  | 0.000 |
| Excellent | 358 | 6.1 | 295 | 6.6 |  |
| Very good | 1942 | 33.3 | 1653 | 36.9 |  |
| Good | 2770 | 47.5 | 2053 | 45.8 |  |
| Regular | 688 | 11.8 | 433 | 9.7 |  |
| Bad | 79 | 1.4 | 47 | 1.0 |  |
| Total | 5837 | 100.0 | 4481 | 100,0 |  |
| **Chronic disease** |  |  |  |  | 0.307 |
| No | 3836 | 66.1 | 2790 | 65.1 |  |
| Yes | 1967 | 33.9 | 1494 | 34.9 |  |
| Total | 5803 | 100.0 | 4284 | 100,0 |  |
| **Baseline smoking status (before confinement)** |  |  |  |  | 0.001 |
| non-smoker | 4968 | 82.2 | 3928 | 84.7 |  |
| smoker | 1075 | 17.8 | 711 | 15.3 |  |
| Total | 6043 | 100.0 | 4639 | 100.0 |  |
| **BMI status (before confinement)** |  |  |  |  | 0.44 |
| under/normal | 2689 | 44.3 | 2084 | 45.1 |  |
| overweight/obese | 3381 | 55.7 | 2542 | 54.9 |  |
| Total | 6070 | 100.0 | 4626 | 100.0 |  |

**Supplementary Resource 2.** Contribution of each isotype-antigen combination to the overall SARS-CoV-2 serostatus (negative, undetermined, positive).

| **Supplementary Resource 3.** Seroprevalence among participants of the first and the second sampling period. | | | | | | | | | | | | | | |
| --- | --- | --- | --- | --- | --- | --- | --- | --- | --- | --- | --- | --- | --- | --- |
|  | First sampling period (n=3651) | | | | | |  | Second sampling period (n=1089) | | | | | |  |
|  | positive | | negative | | undetermined | |  | positive | | negative | | undetermined | |  |
|  | n | % | n | % | n | % |  | n | % | n | % | n | % | p-value |
| overall | 599 | 16.4 | 2665 | 73.0 | 387 | 10.6 |  | 259 | 23.8 | 666 | 61.2 | 164 | 15.1 | 0.000 |
| by isotype |  |  |  |  |  |  |  |  |  |  |  |  |  |  |
| IgM | 118 | 3.2 | 3422 | 93.7 | 111 | 3.0 |  | 57 | 5.2 | 970 | 89.1 | 62 | 5.7 | 0.000 |
| IgA | 496 | 13.6 | 2847 | 78.0 | 308 | 8.4 |  | 198 | 18.2 | 769 | 70.6 | 122 | 11.2 | 0.000 |
| IgG | 303 | 8.3 | 3239 | 88.7 | 109 | 3.0 |  | 123 | 11.3 | 895 | 82.2 | 71 | 6.5 | 0.000 |
| by isotype-antigen combination |  |  |  |  |  |  |  |  |  |  |  |  |  |  |
| IgM NFL | 4 | 0.1 | 3622 | 99.2 | 25 | 0.7 |  | 2 | 0.2 | 1072 | 98.4 | 15 | 1.4 | 0.075 |
| IgM NCt | 10 | 0.3 | 3619 | 99.1 | 22 | 0.6 |  | 8 | 0.7 | 1055 | 96.9 | 26 | 2.4 | 0.000 |
| IgM RBD | 58 | 1.6 | 3522 | 96.5 | 71 | 1.9 |  | 30 | 2.8 | 1030 | 94.6 | 29 | 2.7 | 0.014 |
| IgM S | 30 | 0.8 | 3553 | 97.3 | 68 | 1.9 |  | 19 | 1.7 | 1048 | 96.2 | 22 | 2.0 | 0.028 |
| IgM S2 | 14 | 0.4 | 3578 | 98.0 | 59 | 1.6 |  | 6 | 0.6 | 1059 | 97.2 | 24 | 2.2 | 0.323 |
| IgA NFL | 87 | 2.4 | 3355 | 91.9 | 209 | 5.7 |  | 53 | 4.9 | 955 | 87.7 | 81 | 7.4 | 0.000 |
| IgA NCt | 72 | 2.0 | 3511 | 96.2 | 68 | 1.9 |  | 57 | 5.2 | 968 | 88.9 | 64 | 5.9 | 0.000 |
| IgA RBD | 248 | 6.8 | 3261 | 89.3 | 142 | 3.9 |  | 90 | 8.3 | 949 | 87.1 | 50 | 4.6 | 0.134 |
| IgA S | 237 | 6.5 | 3305 | 90.5 | 109 | 3.0 |  | 88 | 8.1 | 954 | 87.6 | 47 | 4.3 | 0.015 |
| IgA S2 | 154 | 4.2 | 3255 | 89.2 | 242 | 6.6 |  | 55 | 5.1 | 946 | 86.9 | 88 | 8.1 | 0.113 |
| IgG NFL | 35 | 1.0 | 3471 | 95.1 | 145 | 4.0 |  | 24 | 2.2 | 1002 | 92.0 | 63 | 5.8 | 0.000 |
| IgG NCt | 63 | 1.7 | 3468 | 95.0 | 120 | 3.3 |  | 35 | 3.2 | 1013 | 93.0 | 41 | 3.8 | 0.007 |
| IgG RBD | 275 | 7.5 | 3348 | 91.7 | 28 | 0.8 |  | 105 | 9.6 | 960 | 88.2 | 24 | 2.2 | 0.000 |
| IgG S | 258 | 7.1 | 3338 | 91.4 | 55 | 1.5 |  | 93 | 8.5 | 969 | 89.0 | 27 | 2.5 | 0.022 |
| IgG S2 | 66 | 1.8 | 3371 | 92.3 | 214 | 5.9 |  | 38 | 3.5 | 982 | 90.2 | 69 | 6.3 | 0.003 |
| First sampling period: June 23 to July 31, 2020; Second sampling period: September 8 to November 17, 2020. | | | | | | | | | | | | | | |

| **Supplementary Resource 4.** Seroprevalence among the 99 participants with a previously positive SARS-CoV-2 test. | | | | | | |
| --- | --- | --- | --- | --- | --- | --- |
|  | positive | | negative | | undetermined | |
|  | n | % | n | % | n | % |
| Total | 91 | 91.9 | 7 | 7.1 | 1 | 1.0 |
| Isotype |  |  |  |  |  |  |
| IgM | 41 | 41.4 | 44 | 44.4 | 14 | 14.1 |
| IgA | 82 | 82.8 | 10 | 10.1 | 7 | 7.1 |
| IgG | 84 | 84.8 | 13 | 13.1 | 2 | 2.0 |
| Isotype-antigen combination |  |  |  |  |  |  |
| IgM NFL | 1 | 1.0 | 94 | 94.9 | 4 | 4.0 |
| IgM NCt | 0 | 0 | 95 | 96.0 | 4 | 4.0 |
| IgM RBD | 29 | 29.3 | 58 | 58.6 | 12 | 12.1 |
| IgM S | 18 | 18.2 | 59 | 59.6 | 22 | 22.2 |
| IgM S2 | 6 | 6.1 | 77 | 77.8 | 16 | 16.2 |
| IgA NFL | 18 | 18.2 | 56 | 56.6 | 25 | 25.3 |
| IgA NCt | 15 | 15.2 | 75 | 75.8 | 9 | 9.1 |
| IgA RBD | 66 | 66.7 | 18 | 18.2 | 15 | 15.2 |
| IgA S | 70 | 70.7 | 19 | 19.2 | 10 | 10.1 |
| IgA S2 | 45 | 45.5 | 31 | 31.3 | 23 | 23.2 |
| IgG NFL | 23 | 23.2 | 41 | 41.4 | 35 | 35.4 |
| IgG NCt | 31 | 31.3 | 39 | 39.4 | 29 | 29.3 |
| IgG RBD | 84 | 84.8 | 15 | 15.2 | 0 | 0 |
| IgG S | 81 | 81.8 | 16 | 16.2 | 2 | 2.0 |
| IgG S2 | 33 | 33.3 | 16 | 16.2 | 50 | 50.5 |

| **Supplementary Resource 5.** Characteristics by serostatus and then by severity among seropositive participants. | | | | | | | | | | | | | | | |
| --- | --- | --- | --- | --- | --- | --- | --- | --- | --- | --- | --- | --- | --- | --- | --- |
| Characteristic | Seronegatives/undetermined (n=3882) | | Seropositives (n=858) | |  |  | Seropositives by severity of infection | | | | | | | |  |
|  |  |  |  |  |  |  | Asymptomatics (n=322) | | 1-3 symptoms (n=276) | | ≥4 symptoms (n=216) | | admitted to hospital/ICU (n=24) | |  |
|  | n | % | n | % | p-value |  | n | % | n | % | n | % | n | % | p-value |
| **Age** |  |  |  |  | 0.62 |  |  |  |  |  |  |  |  |  | 0.000 |
| 20-40 years old | 143 | 3.7 | 32 | 3.8 |  |  | 9 | 2.8 | 11 | 4.0 | 5 | 2.3 | 0 | 0 |  |
| 40-60 years old | 2564 | 67.0 | 555 | 65.5 |  |  | 179 | 55.6 | 183 | 66.3 | 176 | 81.5 | 15 | 62.5 |  |
| 60-80 years old | 1098 | 28.7 | 257 | 30.3 |  |  | 133 | 41.3 | 80 | 29.0 | 35 | 16.2 | 9 | 37.5 |  |
| 80-93 years old | 24 | 0.6 | 3 | 0.4 |  |  | 1 | 0.3 | 2 | 0.7 | 0 | 0 | 0 | 0 |  |
| total | 3829 | 100.0 | 847 | 100.0 |  |  | 322 | 100.0 | 276 | 100.0 | 216 | 100.0 | 24 | 100.0 |  |
| **Gender** |  |  |  |  | 0.68 |  |  |  |  |  |  |  |  |  | 0.001 |
| Male | 1491 | 38.4 | 323 | 37.6 |  |  | 146 | 45.3 | 104 | 37.7 | 60 | 27.8 | 11 | 45.8 |  |
| Female | 2391 | 61.6 | 535 | 62.4 |  |  | 176 | 54.7 | 172 | 62.3 | 156 | 72.2 | 13 | 54.2 |  |
| Total | 3882 | 100.0 | 858 | 100.0 |  |  | 322 | 100.0 | 276 | 100.0 | 216 | 100.0 | 24 | 100.0 |  |
| **Educational level** |  |  |  |  | 0.19 |  |  |  |  |  |  |  |  |  | 0.478 |
| Primary or less | 417 | 10.9 | 94 | 11.1 |  |  | 44 | 13.7 | 25 | 9.1 | 21 | 9.7 | 4 | 16.7 |  |
| Secondary | 1539 | 40.2 | 345 | 40.7 |  |  | 128 | 39.8 | 112 | 40.6 | 92 | 42.6 | 12 | 50.0 |  |
| University | 1862 | 48.7 | 403 | 47.6 |  |  | 148 | 46.0 | 136 | 49.3 | 103 | 47.7 | 8 | 33.3 |  |
| Other | 7 | 0.2 | 5 | 0.6 |  |  | 2 | 0.6 | 3 | 1.1 | 0 | 0 | 0 | 0 |  |
| Total | 3825 | 100.0 | 847 | 100.0 |  |  | 322 | 100.0 | 276 | 100.0 | 216 | 100.0 | 24 | 100.0 |  |
| **degree of urbanization** |  |  |  |  | 0.85 |  |  |  |  |  |  |  |  |  | 0.487 |
| city | 3149 | 91.0 | 691 | 91.4 |  |  | 260 | 89.3 | 223 | 91.4 | 186 | 93.9 | 22 | 95.7 |  |
| town/suburb | 270 | 7.8 | 55 | 7.3 |  |  | 27 | 9.3 | 16 | 6.6 | 11 | 5.6 | 1 | 4.3 |  |
| rural area | 41 | 1.2 | 10 | 1.3 |  |  | 4 | 1.4 | 5 | 2.0 | 1 | 0.5 | 0 | 0 |  |
| Total | 3460 | 100.0 | 756 | 100.0 |  |  | 291 | 100.0 | 244 | 100.0 | 198 | 100.0 | 23 | 100.0 |  |
| **SARS-COV-2 test previously** |  |  |  |  | 0.00 |  |  |  |  |  |  |  |  |  | 0.000 |
| no | 3169 | 86.0 | 622 | 76.4 |  |  | 259 | 83.0 | 219 | 82.6 | 142 | 66.7 | 0 | 0 |  |
| yes | 515 | 14.0 | 192 | 23.6 |  |  | 53 | 17.0 | 46 | 17.4 | 71 | 33.3 | 24 | 100.0 |  |
| Total | 3684 | 100.0 | 814 | 100.0 |  |  | 312 | 100.0 | 265 | 100.0 | 213 | 100.0 | 24 | 100.0 |  |
| **Contact with a COVID-19 case** |  |  |  |  | 0.000 |  |  |  |  |  |  |  |  |  | 0.000 |
| no | 2531 | 67.6 | 468 | 56.5 |  |  | 225 | 70.8 | 152 | 55.9 | 84 | 39.1 | 7 | 29.2 |  |
| yes | 544 | 14.5 | 207 | 25.0 |  |  | 43 | 13.5 | 76 | 27.9 | 77 | 35.8 | 11 | 45.8 |  |
| don't know | 669 | 17.9 | 154 | 18.6 |  |  | 50 | 15.7 | 44 | 16.2 | 54 | 25.1 | 6 | 25.0 |  |
| Total | 3744 | 100.0 | 829 | 100.0 |  |  | 318 | 100.0 | 272 | 100.0 | 215 | 100.0 | 24 | 100.0 |  |
| **Work during confinement** |  |  |  |  | 0.06 |  |  |  |  |  |  |  |  |  | 0.097 |
| No | 1476 | 40.8 | 342 | 42.8 |  |  | 144 | 46.8 | 108 | 41.4 | 75 | 36.2 | 15 | 65.2 |  |
| Yes, teleworking | 1166 | 32.2 | 219 | 27.4 |  |  | 79 | 25.6 | 71 | 27.2 | 67 | 32.4 | 2 | 8.7 |  |
| Yes, partially teleworking | 320 | 8.8 | 81 | 10.1 |  |  | 28 | 9.1 | 27 | 10.3 | 22 | 10.6 | 4 | 17.4 |  |
| Yes, in my usual place of work | 654 | 18.1 | 157 | 19.6 |  |  | 57 | 18.5 | 55 | 21.1 | 43 | 20.8 | 2 | 8.7 |  |
| Total | 3616 | 100.0 | 799 | 100.0 |  |  | 308 | 100.0 | 261 | 100.0 | 207 | 100.0 | 23 | 100.0 |  |
| **Chronic disease (any)** |  |  |  |  | 0.918 |  |  |  |  |  |  |  |  |  | 0.005 |
| No | 2286 | 65.1 | 504 | 65.3 |  |  | 209 | 70.4 | 168 | 67.5 | 116 | 57.1 | 11 | 47.8 |  |
| Yes | 1226 | 34.9 | 268 | 34.7 |  |  | 88 | 29.6 | 81 | 32.5 | 87 | 42.9 | 12 | 52.2 |  |
| Total | 3512 | 100.0 | 772 | 100.0 |  |  | 297 | 100.0 | 249 | 100.0 | 203 | 100.0 | 23 | 100.0 |  |
| **BMI status (before lockdown)** |  |  |  |  | 0.071 |  |  |  |  |  |  |  |  |  | 0.035 |
| under/normal | 1730 | 45.7 | 354 | 42.2 |  |  | 133 | 41.4 | 132 | 48.2 | 82 | 38.0 | 6 | 25.0 |  |
| overweight/obese | 2058 | 54.3 | 484 | 57.8 |  |  | 188 | 58.6 | 142 | 51.8 | 134 | 62.0 | 18 | 75.0 |  |
| Total | 3788 | 100.0 | 838 | 100.0 |  |  | 321 | 100.0 | 274 | 100.0 | 216 | 100.0 | 24 | 100.0 |  |
| **Smoking status (before lockdown)** |  |  |  |  | 0.047 |  |  |  |  |  |  |  |  |  | 0.108 |
| non-smoker | 3198 | 84.2 | 730 | 86.9 |  |  | 272 | 84.7 | 236 | 86.4 | 192 | 89.3 | 24 | 100.0 |  |
| smoker | 601 | 15.8 | 110 | 13.1 |  |  | 49 | 15.3 | 37 | 13.6 | 23 | 10.7 | 0 | 0 |  |
| Total | 3799 | 100.0 | 840 | 100.0 |  |  | 321 | 100.0 | 273 | 100.0 | 215 | 100.0 | 24 | 100.0 |  |
|  |  |  |  |  |  |  | mean (SD) |  | mean (SD) |  | mean (SD) |  | mean (SD) |  |  |
| **Days since infection** |  |  |  |  |  |  | 149 (46) |  | 135 (56) |  | 143 (44) |  | 140 (52) |  | 0.007* |
| p-values are based on x2 test and Fisher's exact test for sparse data; | | | | | | | | | | | | | | | |
| * p-value is based on a t-test, pairwise comparisons with Tukey post hoc test identified statistical significant differences only between asymptomatics and those with 1-3 symptoms | | | | | | | | | | | | | | | |

| **Supplementary Resource 6**. P-values for comparisons related to Figure 2. | | | | | | | |
| --- | --- | --- | --- | --- | --- | --- | --- |
| Immune variable | oneway anova | Pair-wise comparisons Tukeypost-hoc tests | | | | | |
|  |  | 1-3symptoms | ≥4 symptoms | hospital/ICU | ≥4 symptoms | hospital/ICU | hospital/ICU |
|  |  | asymptomatics | asymptomatics | asymptomatics | 1-3symptoms | 1-3symptoms | ≥4 symptoms |
| **Figure 2a** |  |  |  |  |  |  |  |
| IgM NFL | 0.7995 | 0.804 | 0.996 | 0.950 | 0.933 | 0.998 | 0.972 |
| IgM NCt | 0.9296 | 0.989 | 1.000 | 0.924 | 0.997 | 0.959 | 0.938 |
| IgM RBD | 0.0000 | 0.777 | 0.0000 | 0.0000 | 0.0000 | 0.0000 | 0.004 |
| IgM S | 0.0000 | 0.629 | 0.0000 | 0.0000 | 0.0000 | 0.0000 | 0.007 |
| IgM S2 | 0.0000 | 0.131 | 0.0000 | 0.0000 | 0.0000 | 0.001 | 0.387 |
| IgA NFL | 0.0147 | 0.968 | 0.998 | 0.012 | 0.995 | 0.007 | 0.011 |
| IgA NCt | 0.0102 | 0.456 | 0.366 | 0.079 | 0.995 | 0.017 | 0.014 |
| IgA RBD | 0.0000 | 0.089 | 0.0000 | 0.0000 | 0.0000 | 0.0000 | 0.0000 |
| IgA S | 0.0000 | 0.065 | 0.0000 | 0.0000 | 0.0000 | 0.0000 | 0.0000 |
| IgA S2 | 0.0000 | 0.089 | 0.0000 | 0.0000 | 0.0000 | 0.0000 | 0.003 |
| IgG NFL | 0.0000 | 0.002 | 0.0000 | 0.0000 | 0.0000 | 0.0000 | 0.002 |
| IgG NCt | 0.0000 | 0.0000 | 0.0000 | 0.0000 | 0.0000 | 0.0000 | 0.0000 |
| IgG RBD | 0.0000 | 0.0000 | 0.0000 | 0.0000 | 0.0000 | 0.0000 | 0.0000 |
| IgG S | 0.0000 | 0.0000 | 0.0000 | 0.0000 | 0.0000 | 0.0000 | 0.0000 |
| IgG S2 | 0.0000 | 0.001 | 0.0000 | 0.0000 | 0.0000 | 0.0000 | 0.001 |
| **Figure 2b** |  |  |  |  |  |  |  |
| breadth | 0.0000 | 0.001 | 0.0000 | 0.0000 | 0.0000 | 0.0000 | 0.0000 |
| **Figure 2c** |  |  |  |  |  |  |  |
| RBD IgA/IgG | 0.0000 | 0.0000 | 0.0000 | 0.0000 | 0.0000 | 0.0000 | 0.174 |
| S IgA/IgG | 0.0000 | 0.002 | 0.0000 | 0.0000 | 0.0000 | 0.002 | 0.445 |
| S2 IgA/IgG | 0.2789 | 0.775 | 0.22 | 0.894 | 0.751 | 0.987 | 1 |
| NFL IgA/IgG | 0.0000 | 0.06 | 0.0000 | 0.01 | 0.0000 | 0.137 | 0.999 |
| NCt IgA/IgG | 0.0000 | 0.0000 | 0.0000 | 0.0000 | 0.0000 | 0.001 | 0.507 |
| **Figure 2e** |  |  |  |  |  |  |  |
| IgA RBD/NFL | 0.0000 | 0.168 | 0.0000 | 0.001 | 0.0000 | 0.023 | 0.837 |
| IgA RBD/NCt | 0.0000 | 0.009 | 0.0000 | 0.0000 | 0.0000 | 0.0000 | 0.136 |
| IgA S/NCt | 0.0000 | 0.007 | 0.0000 | 0.0000 | 0.0000 | 0.0000 | 0.328 |
| IgA S/NFL | 0.0000 | 0.105 | 0.0000 | 0.0000 | 0.0000 | 0.005 | 0.919 |
| IgA S2/NCt | 0.0000 | 0.016 | 0.0000 | 0.0000 | 0.0000 | 0.0000 | 0.687 |
| IgA S2/NFL | 0.0000 | 0.093 | 0.0000 | 0.002 | 0.0000 | 0.035 | 0.994 |
| IgM RBD/NCt | 0.0000 | 0.738 | 0.0000 | 0.0000 | 0.0000 | 0.0000 | 0.001 |
| IgM RBD/NFL | 0.0000 | 0.508 | 0.0000 | 0.0000 | 0.0000 | 0.0000 | 0.002 |
| IgM S/NCt | 0.0000 | 0.586 | 0.0000 | 0.0000 | 0.0000 | 0.0000 | 0.001 |
| IgM S/NFL | 0.0000 | 0.354 | 0.0000 | 0.0000 | 0.0000 | 0.0000 | 0.004 |
| IgM S2/NCt | 0.0000 | 0.086 | 0.0000 | 0.0000 | 0.0000 | 0.0000 | 0.172 |
| IgM S2/NFL | 0.0000 | 0.045 | 0.0000 | 0.0000 | 0.0000 | 0.001 | 0.274 |
| IgG RBD/NFL | 0.0000 | 0.0000 | 0.0000 | 0.0000 | 0.0000 | 0.0000 | 0.0000 |
| IgG RBD/NCt | 0.0000 | 0.007 | 0.0000 | 0.0000 | 0.0000 | 0.0000 | 0.041 |
| IgG S/NCt | 0.0000 | 0.068 | 0.0000 | 0.0000 | 0.0000 | 0.0000 | 0.374 |
| IgG S/NFL | 0.0000 | 0.003 | 0.0000 | 0.0000 | 0.0000 | 0.0000 | 0.017 |
| IgG S2/NCt | 0.0186 | 0.961 | 0.045 | 0.989 | 0.016 | 0.958 | 0.874 |
| IgG S2/NFL | 0.0037 | 0.918 | 0.008 | 0.177 | 0.058 | 0.287 | 0.900 |
| sum of S>N features | 0.0000 | 0.008 | 0.0000 | 0.0000 | 0.0000 | 0.0000 | 0.035 |

| **Supplementary Resource 7.** Associations (β, 95% CI) between each characteristic and antibody levels (log10 transformed) for each of the 15 isotype-antigen combinations as well as the breadth of positive immune responses among SARS-CoV-2 seropositive participants. | | | | | | | | | | | | | | | |
| --- | --- | --- | --- | --- | --- | --- | --- | --- | --- | --- | --- | --- | --- | --- | --- |
|  | IgM NFL | | | IgM NCt | | IgM RBD | | IgM S | | | IgM S2 | | |  |  |
| Characteristic | β | 95% CI | | β | 95% CI | β | 95% CI | β | 95% CI | | β | 95% CI | |  |  |
| Αbove 60 years old | -0.02 | [-0.08,0.04] | | -0.02 | [-0.08,0.03] | -0.10** | [-0.16,-0.03] | -0.07* | [-0.14,-0.01] | | -0.12*** | [-0.19,-0.06] | |  |  |
| Female sex | 0.15*** | [0.09,0.21] | | 0.12*** | [0.07,0.17] | 0.03 | [-0.03,0.09] | 0.04 | [-0.02,0.10] | | 0.07* | [0.00,0.13] | |  |  |
| Obese/overweight | -0.02 | [-0.08,0.04] | | 0.01 | [-0.04,0.06] | 0.05 | [-0.01,0.12] | 0.05 | [-0.01,0.11] | | 0.02 | [-0.04,0.08] | |  |  |
| Smoker | 0.02 | [-0.07,0.10] | | -0.00 | [-0.07,0.07] | -0.05 | [-0.14,0.04] | -0.09* | [-0.18,-0.01] | | -0.10* | [-0.19,-0.01] | |  |  |
| *After adjustmenet for severity of infection* |  |  | |  |  |  |  |  |  | |  |  | |  |  |
| above 60 years old | -0.03 | [-0.09,0.04] | | -0.03 | [-0.08,0.02] | -0.04 | [-0.11,0.02] | -0.02 | [-0.08,0.04] | | -0.08* | [-0.14,-0.02] | |  |  |
| Female sex | 0.16*** | [0.10,0.22] | | 0.12*** | [0.08,0.17] | -0.00 | [-0.06,0.06] | 0.01 | [-0.05,0.06] | | 0.04 | [-0.02,0.10] | |  |  |
| Obese/overweight | -0.01 | [-0.07,0.04] | | 0.02 | [-0.03,0.06] | 0.03 | [-0.03,0.09] | 0.03 | [-0.03,0.09] | | 0.00 | [-0.06,0.06] | |  |  |
| Smoker | 0.01 | [-0.07,0.10] | | -0.00 | [-0.08,0.07] | -0.01 | [-0.10,0.07] | -0.06 | [-0.14,0.03] | | -0.07 | [-0.15,0.02] | |  |  |
| *Excluding seronegatives for each isotype-antigen combination examined* | *(n=34)* |  | | *(n=43)* |  | *(n=163)* |  | *(n=123)* |  | | *(n=77)* |  | |  |  |
| above 60 years old | 0.03 | [-0.16,0.22] | | -0.00 | [-0.18,0.17] | 0.09 | [-0.05,0.23] | 0.07 | [-0.08,0.21] | | -0.05 | [-0.21,0.11] | |  |  |
| Female sex | -0.05 | [-0.29,0.19] | | -0.09 | [-0.27,0.09] | -0.10 | [-0.22,0.03] | **-0.14*** | [-0.27,-0.01] | | -0.12 | [-0.27,0.04] | |  |  |
| Obese/overweight | -0.16 | [-0.35,0.02] | | 0.11 | [-0.04,0.27] | **0.14*** | [0.02,0.26] | 0.10 | [-0.03,0.23] | | 0.06 | [-0.07,0.20] | |  |  |
| Smoker | -0.03 | [-0.24,0.19] | | -0.02 | [-0.20,0.16] | 0.14 | [-0.04,0.33] | -0.05 | [-0.28,0.19] | | 0.04 | [-0.26,0.34] | |  |  |
|  | IgA NFL | | | IgA NCt | | IgA RBD | | IgA S | | | IgA S2 | | |  |  |
| Characteristic | β | | 95% CI | β | 95% CI | β | 95% CI | β | | 95% CI | β | | 95% CI |  |  |
| Αbove 60 years old | 0.10* | | [0.02,0.18] | 0.00 | [-0.05,0.06] | -0.07** | [-0.12,-0.02] | -0.09** | | [-0.15,-0.02] | -0.13** | | [-0.22,-0.04] |  |  |
| Female sex | -0.05 | | [-0.12,0.03] | -0.04 | [-0.09,0.01] | -0.01 | [-0.06,0.04] | -0.00 | | [-0.07,0.06] | -0.09* | | [-0.18,-0.01] |  |  |
| Obese/overweight | 0.07 | | [-0.01,0.14] | 0.04 | [-0.01,0.09] | 0.11*** | [0.06,0.16] | 0.08* | | [0.02,0.14] | 0.10* | | [0.02,0.18] |  |  |
| Smoker | -0.05 | | [-0.16,0.06] | -0.02 | [-0.09,0.05] | -0.10** | [-0.17,-0.03] | -0.16*** | | [-0.25,-0.07] | -0.17** | | [-0.29,-0.05] |  |  |
| *After adjustmenet for severity of infection* |  | |  |  |  |  |  |  | |  |  | |  |  |  |
| above 60 years old | 0.11** | | [0.03,0.19] | 0.00 | [-0.05,0.05] | -0.02 | [-0.07,0.03] | -0.02 | | [-0.08,0.04] | -0.04 | | [-0.13,0.04] |  |  |
| Female sex | -0.05 | | [-0.13,0.02] | -0.04 | [-0.09,0.01] | -0.04 | [-0.09,0.00] | -0.04 | | [-0.10,0.02] | -0.14*** | | [-0.22,-0.06] |  |  |
| Obese/overweight | 0.06 | | [-0.01,0.14] | 0.04 | [-0.01,0.09] | 0.09*** | [0.04,0.13] | 0.05 | | [-0.00,0.11] | 0.07 | | [-0.01,0.14] |  |  |
| Smoker | -0.05 | | [-0.15,0.06] | -0.02 | [-0.09,0.05] | -0.06 | [-0.13,0.00] | -0.11** | | [-0.20,-0.03] | -0.11 | | [-0.22,0.00] |  |  |
| *Excluding seronegatives for each isotype-antigen combination examined* | *(n=309)* | |  | *(n=223)* |  | *(n=452)* |  | *(n=419)* | |  | *(n=401)* | |  |  |  |
| above 60 years old | -0.01 | | [-0.09,0.08] | -0.07 | [-0.16,0.03] | -0.05 | [-0.12,0.02] | -0.04 | | [-0.12,0.04] | **-0.10**** | | [-0.18,-0.02] |  |  |
| Female sex | 0.03 | | [-0.06,0.11] | **-0.10*** | [-0.19,-0.00] | -0.02 | [-0.09,0.04] | -0.02 | | [-0.09,0.06] | 0.01 | | [-0.06,0.08] |  |  |
| Obese/overweight | 0.01 | | [-0.07,0.10] | 0.03 | [-0.07,0.13] | **0.09**** | [0.02,0.16] | 0.04 | | [-0.03,0.12] | 0.02 | | [-0.05,0.09] |  |  |
| Smoker | -0.08 | | [-0.22,0.05] | 0.07 | [-0.10,0.24] | **-0.11*** | [-0.21,-0.01] | -0.09 | | [-0.22,0.03] | -0.10 | | [-0.22,0.01] |  |  |
|  | IgG NFL | | | IgG NCt | | IgG RBD | | IgG S | | | IgG S2 | | | Breadth | |
| Characteristic | β | | 95% CI | β | 95% CI | β | 95% CI | β | | 95% CI | β | | 95% CI | β | 95% CI |
| Αbove 60 years old | -0.03 | | [-0.11,0.05] | -0.10* | [-0.19,-0.02] | -0.17** | [-0.29,-0.05] | -0.15* | | [-0.27,-0.03] | -0.14** | | [-0.24,-0.04] | -0.46* | [-0.88,-0.03] |
| Female sex | 0.03 | | [-0.04,0.11] | -0.04 | [-0.12,0.04] | 0.04 | [-0.08,0.15] | 0.02 | | [-0.10,0.13] | -0.04 | | [-0.13,0.06] | -0.09 | [-0.50,0.31] |
| Obese/overweight | 0.08* | | [0.01,0.16] | 0.09* | [0.02,0.17] | 0.17** | [0.06,0.28] | 0.16** | | [0.05,0.28] | 0.13** | | [0.03,0.22] | 0.77*** | [0.37,1.17] |
| Smoker | -0.23*** | | [-0.34,-0.12] | -0.21*** | [-0.32,-0.09] | -0.36*** | [-0.53,-0.20] | -0.39*** | | [-0.56,-0.22] | -0.29*** | | [-0.42,-0.15] | -1.17*** | [-1.75,-0.58] |
| *After adjustmenet for severity of infection* |  | |  |  |  |  |  |  | |  |  | |  |  |  |
| above 60 years old | 0.06 | | [-0.01,0.13] | -0.02 | [-0.09,0.06] | -0.01 | [-0.12,0.09] | 0.00 | | [-0.10,0.11] | -0.02 | | [-0.11,0.07] | 0.07 | [-0.31,0.45] |
| Female sex | -0.02 | | [-0.09,0.05] | -0.09* | [-0.16,-0.02] | -0.05 | [-0.15,0.05] | -0.07 | | [-0.17,0.03] | -0.10* | | [-0.19,-0.02] | -0.39* | [-0.75,-0.03] |
| Obese/overweight | 0.05 | | [-0.02,0.12] | 0.06 | [-0.01,0.13] | 0.11* | [0.01,0.20] | 0.10 | | [-0.00,0.20] | 0.08 | | [-0.00,0.17] | 0.56** | [0.21,0.92] |
| Smoker | -0.17** | | [-0.27,-0.07] | -0.14** | [-0.25,-0.04] | -0.25*** | [-0.39,-0.11] | -0.28*** | | [-0.43,-0.13] | -0.20** | | [-0.33,-0.08] | -0.79** | [-1.31,-0.27] |
| *Excluding seronegatives for each isotype-antigen combination examined* | *(n=239)* | |  | *(n=241)* |  | *(n=394)* |  | *(n=379)* | |  | *(n=354)* | |  |  |  |
| above 60 years old | 0.01 | | [-0.05,0.06] | 0.05 | [-0.03,0.13] | 0.03 | [-0.08,0.15] | 0.02 | | [-0.07,0.10] | 0.04 | | [-0.00,0.09] |  |  |
| Female sex | **-0.08**** | | [-0.13,-0.03] | -0.04 | [-0.12,0.03] | -0.04 | [-0.14,0.07] | 0.02 | | [-0.05,0.10] | -0.01 | | [-0.05,0.04] |  |  |
| Obese/overweight | **0.08**** | | [0.02,0.13] | 0.06 | [-0.01,0.14] | **0.16**** | [0.05,0.26] | **0.10*** | | [0.02,0.18] | **0.09***** | | [0.05,0.13] |  |  |
| Smoker | -0.05 | | [-0.15,0.04] | -0.07 | [-0.21,0.07] | **-0.33***** | [-0.50,-0.15] | **-0.18**** | | [-0.32,-0.05] | **-0.11**** | | [-0.18,-0.03] |  |  |
| All models are linear regression models adjusted forall the listed characteristics and days since infection | | | | | | | | | | | | | | | |
| * p-value<0.05, ** p-value<0.01, *** p-value<0.001 | | | | | | | | | | | | | | | |

| **Supplementary Resource 8.** Seroresponses to each of the 15 isotype-antigen combinations among seropositive teenagers and parents of the INMA mother-child cohort. | | | |
| --- | --- | --- | --- |
| Isotype-antigen combination | Teenagers (n=30) | Parents (n=50) |  |
|  | mean (SD) | mean (SD) | p-value |
| IgM N-FL | 3.02 (0.30) | 3.18 (0.38) | 0.0541 |
| IgM N-Ct | 2.70 (0.28) | 2.82 (0.36) | 0.1431 |
| IgM RBD | 2.62 (0.24) | 2.66 (0.32) | 0.5239 |
| IgM S | 2.53 (0.22) | 2.55 (0.26) | 0.82 |
| IgM S2 | 3.07 (0.29) | 3.04 (0.39) | 0.71 |
| IgA N-FL | 2.74 (0.32) | 3.17 (0.41) | 0.00 |
| IgA N-Ct | 2.50 (0.12) | 2.71 (0.21) | 0.00 |
| IgA RBD | 2.54 (0.25) | 2.50 (0.26) | 0.4345 |
| IgA S | 2.56 (0.32) | 2.53 (0.31) | 0.6968 |
| IgA S2 | 3.13 (0.53) | 3.14 (0.48) | 0.9119 |
| IgG N-FL | 4.24 (0.52) | 4.08 (0.49) | 0.1776 |
| IgG N-Ct | 3.68 (0.55) | 3.49 (0.47) | 0.1086 |
| IgG RBD | 3.56 (0.77) | 3.08 (0.67) | 0.0041 |
| IgG S | 3.82 (0.73) | 3.37 (0.72) | 0.0093 |
| IgG S2 | 4.50 (0.58) | 4.09 (0.68) | 0.0073 |
| p-value based on t-test | | | |

| **Supplementary Resource 9.** Description of variables, sources and methods of assessment. | | |
| --- | --- | --- |
| Variable name | Sources of data | Specify measure if not standard |
| **Age of the participant** | COVICAT questionnaire, self-reported | Recoded as 20-40, 40-60, 60-80, 80-93 years old (adolescents are analyzed separately) |
|  |  | Recoded as >60 years old: yes or no (People aged 60 years and older are high risk group for COVID-19. This categorization was used to explore the effect of age , being over 60 years or older, on antibody levels among seropositive participants) |
| **Gender** | COVICAT questionnaire, self-reported | Categories: male, female |
| **Ethnicity** | Data from previous information of the cohorts | Categories: white/Caucasian, other |
| **Degree of urbanization** | Data from previous information of the cohorts | Recoded as city, town or suburb, rural area. The boundaries of the degree of urbanization areas are derived from the LAU2 2018 boundaries from the European Commission - Eurostat/GISCO, based on data from EuroGeographics and the Eurostat 2011 Population grid. https://ec.europa.eu/eurostat/web/degree-of-urbanisation/background |
| **Health region** | COVICAT questionnaire | Categories: Alt Pirineu i Aran,Catalunya Central, Girona, Lleida, Terres de l'Ebre, Camp de Tarragona, Ambit Metropolita Sud, Barcelona Ciutat, Ambit Metropolita Nord. Health services in Catalunya are organized in regions to cover social and health services in all the territory (they are not political divisions). |
| **Educational level** | COVICAT questionnaire | Highest attained educational level. Categories: primary or less, secondary, university, other |
| **Work during confinement** | COVICAT questionnaire, not available for INMA | Categories: no, yes teleworking, yes partially teleworking, yes in my usual place of work |
| **Contact with a COVID-19 case** | COVICAT questionnaire | "Have you had contact with a person who was a confirmed COVID-19 case? No, yes, don't know" |
| **Self-reported symptoms compatible with COVID-19** | COVICAT questionnaire, BISC participants: were not asked about muscle/joint pain, runny nose, diarrhea, rash, chest pain, nausea, INMA participants: screening for presence of symptoms with repeated questionnaires every 2 weeks | "Did you have any of these symptoms from February 2020 to date of interview? Fever, cough, dyspnea, loss of odor/taste, headache, muscle/joint pain, fatigue, runny nose, nausea, diarrhea, rash, chest pain" |
| **days since infection** | COVICAT questionnaire | Difference in days between sampling date and date of infection. Date of infection is assumed to be the date of onset of the first symptom. For asymptomatics we defined as date of infection the median date reported among symptomatics. |
| **COVID-19 test** | COVICAT questionnaire | Categories: yes, no |
| **COVID-19 test, result** | COVICAT questionnaire | Categories: positive, negative, don't know |
| **COVID-19 test, date** | COVICAT questionnaire |  |
| **Hospitalized of COVID-19** | COVICAT questionnaire, not available for BISC | Categories: yes, no |
| **Hospital admission date** | COVICAT questionnaire, not available for BISC |  |
| **ICU - intensive care unit** | COVICAT questionnaire, not available for BISC | Categories: yes, no |
| **General health status** | COVICAT questionnaire, not available for BISC | Excellent Very good Good Regular Bad |
| **Smoking status (before lockdown)** | COVICAT questionnaire, based on multiple questions regarding current smoking, smoking changes during confinement and age quitted smoking. For INMA and BISC, it was based from previous information of the cohorts | Categories: non-smokers, smokers. Non-smokers include never smokers and ex-smokers |
| **BMI status (before lockdown)** | COVICAT questionnaire | Recode BMI in categories of underweight/normal weight and overweight/obese using WHO/IOTF cut-offs (we had very few underweight participants, so they were grouped together with normal weight participants). BMI was based on self-reported weight before confinement and current height. |
| **Chronic disease** | COVICAT questionnaire, not available for INMA & BISC | "Do you have any chronic disease (a disease that required medical contact for at least 6 months): yes, no" |
